# Synthesis of (cinnamate-zinc layered hydroxide) intercalation compound for sunscreen application

**DOI:** 10.1186/1752-153X-7-26

**Published:** 2013-02-06

**Authors:** Sumaiyah Megat Nabil Mohsin, Mohd Zobir Hussein, Siti Halimah Sarijo, Sharida Fakurazi, Palanisamy Arulselvan, Taufiq-Yap Yun Hin

**Affiliations:** 1 Material Synthesis and Characterization Laboratory, Institute of Advanced Technology (ITMA), Universiti Putra Malaysia, 43400 UPM, Serdang, Selangor, Malaysia; 2 Faculty of Applied Sciences, Universiti Teknologi MARA, 40450 UiTM, Shah Alam, Selangor, Malaysia; 3 Department of Human Anatomy, Faculty of Medicine and Health Sciences, Universiti Putra Malaysia, 43400 UPM, Serdang, Selangor, Malaysia; 4 Laboratory of Vaccines and Immunotherapeutics, Institute of Bioscience, Universiti Putra Malaysia, 43400 UPM, Serdang, Selangor, Malaysia; 5 Centre of Excellence for Catalysis Science and Technology, Faculty of Science, Universiti Putra Malaysia, 43400 UPM, Serdang, Selangor, Malaysia

**Keywords:** Sunscreen, Zinc layered hydroxide, Zinc oxide, Optical properties, Cytotoxicity

## Abstract

**Background:**

Zinc layered hydroxide (ZLH) intercalated with cinnamate, an anionic form of cinnamic acid (CA), an efficient UVA and UVB absorber, have been synthesized by direct method using zinc oxide (ZnO) and cinnamic acid as the precursor.

**Results:**

The resulting obtained intercalation compound, ZCA, showed a basal spacing of 23.9 Å as a result of cinnamate intercalated in a bilayer arrangement between the interlayer spaces of ZLH with estimated percentage loading of cinnamate of about 40.4 % w/w. The UV–vis absorption spectrum of the intercalation compound showed excellent UVA and UVB absorption ability. Retention of cinnamate in ZLH interlayers was tested against media usually came across with sunscreen usage to show low release over an extended period of time. MTT assay of the intercalation compound on human dermal fibroblast (HDF) cells showed cytotoxicity of ZCA to be concentration dependent and is overall less toxic than its precursor, ZnO.

**Conclusions:**

(Cinnamate-zinc layered hydroxide) intercalation compound is suitable to be used as a safe and effective sunscreen with long UV protection effect.

## Background

World Health Organization estimates that 60,000 deaths occur in 2000 from melanoma and other skin cancers due to solar ultraviolet (UV) radiation [1]. Exposure to sunlight is unavoidable as we go about our daily errands. Sunscreen is our last defense against UV radiation. However, protection with sunscreen deemed inadequate due to photodegradation of organic UV absorber in sunscreen products that not only causes decrease in UV protection but also degrades with the occurrence of toxic degradation products. Therefore, development of a new sunscreen formulation is called for, to avoid previously mentioned effects.

Layered metal hydroxides can be categorized into layered double hydroxide (LDH) and layered hydroxide salt (LHS). Several studies have been done on the use of layered metal hydroxide-based sunscreen carrier system, in particular, LDH [2-4]. LDH can be represented by the general formula of [M^3+^_1-x_M^3+^_x_(OH)_2_]^z+^A^n-^_z/n_.yH_2_O where A^n-^ is the interlayer anion, M^2+^ and M^3+^ are di- and trivalent metallic cation, respectively [2]. Previous work has shown that cinnamate intercalated into Zn/Al LDH showed excellent UV ray shielding properties [4]. However, to the best of our knowledge application of LHS as host has yet to be explored.

Zinc layered hydroxide (ZLH) is a type of LHS, having the general formula of; M^2+^(OH)_2-*x*_(A^*m-*^)_*x/m*_*∙n*H_2_O where M^2+^ in this case is the metal cation Zn^2+^ and A^*m-*^ is the counter ion [5]. They boast a structure consisting of positively charged layers that can expand or contract depending on the nature of interlayer anions. In recent years, there has been extensive research on the use of ZLH as drug carriers [6,7], slow release herbicides [8], flame retardants [9] and anti-corrosion agents [10]. In this study we further exploit its potential as an efficient host to organic molecules for possible application in sunscreen formulation.

In the present work, we investigated the intercalation of anion, cinnamate, into interlayer galleries of ZLH via direct method, as well as properties of the resulting cinnamate-ZLH (ZCA) intercalation compound. Direct method used involved a direct reaction between anion solution and ZnO precursor. Unlike other LHS synthesis methods like hydrolysis of salts and oxides [11], urea hydrolysis [12], precipitation with alkaline solution [13] and solid state reactions [14], this method is simple, environmentally friendly and economical as it involves fewer steps and fewer chemicals.

By intercalating organic UV absorbers into interlayer galleries of ZLH, the expected advantages are; UV absorber stabilization in interlayer region of a lamellar host to increase photo-stability and decrease degradation of UV absorber, absorption of ultraviolet light rays in UVA and UVB region and the absence of close contact between skin that subsequently eliminates allergy problems [15].

In the present investigation, we selected human dermal fibroblast (HDF) cells as a model to evaluate possible toxicity induction on the cells. Dermal fibroblasts are the most abundant cell in the human skin and represent the primary level of exposure to various environmental and other toxicants. Human skin is the primary anatomical barrier for various pathogens and damage, which acts as an important boundary marker between internal and external environment in the bodily defense system. Hence, the resulting intercalation compound of the present study was investigated for toxicity on human dermal fibroblast cells.

## Materials and methods

### Materials

Cinnamic acid (98%) was purchased from Acros (Geel, Belgium). Zinc oxide (99%) was obtained from PC Laboratory chemicals and was used without further purification. Sodium hydroxide (99%) from Merck (Darmstadt, Germany), dimethyl sulfoxide (DMSO) and phosphate-buffered solution from Sigma-Aldrich (Missouri, USA) and sodium chloride (99%) from HmbG Chemicals (Hamburg, Germany) were used without further purifications.

### Synthesis of zinc layered hydroxide intercalated with cinnamate

About 0.2 g of ZnO was reacted with 100 mL of 0.1 mol/L CA solution. The intercalation compound was titrated with 2 mol/L NaOH to the final pH of 8 before it was magnetically stirred for 5 h at room temperature. Then it was aged in an oil bath at 70°C for 18 h, before being centrifuged and washed with deionized water. The final white solid (ZCA) was dried under vacuum at 70°C, overnight.

### Characterization

Powder x-ray diffraction (PXRD) patterns were recorded with a XRD-6000 (Shimazdu, Kyoto, Japan) using CuK_α_ radiation (λ = 1.5418 Å) at 30 kV and 30 mA. The data was collected from 2 - 60º at a dwell time of 0.5º min^-1^. Fourier transform infrared (FTIR) spectra were recorded over the range of 280–4000 cm^-1^ on a Perkin-Elmer Spectrum 100 (Perkin-Elmer, Waltham, Massachusetts, USA) equipped with universal attenuated total reflectance (ATR) accessory. The carbon and hydrogen content in the intercalation compound were analyzed on a CHNS-932 (LECO Instruments, Michigan, USA). The chemical composition of the samples was analyzed for zinc by inductively coupled plasma atomic emission spectrometry (ICP-AES) using a Perkin-Elmer spectrophotometer model Optima 2000DV (Perkin-Elmer, Massachusetts, USA) under standard conditions. Thermogravimetric and differential thermogravimetric analyses (TGA/DTG) were performed on alumina crucibles with a Metter-Toledo instrument model TGA851e (Greifensee, Switzerland) at a heating rate of 10°C min^-1^ in the range of 25 – 1000°C and under nitrogen gas flow of about 50 mL·min^−1^. Surface characterization of the materials was carried out using a nitrogen gas adsorption-desorption technique at 77 K with a Micromeritics, ASAP2000 (Georgia, USA). The surface morphology of the samples was observed by a field emission scanning electron microscopy (FESEM) using a ZEISS supra 40VP (Oberkochen, German), and optical measurements were performed on a Shimadzu (Kyoto, Japan) UV–VIS-NIR diffuse reflectance spectrometer (UV-3600 model).

### Release of cinnamate from ZCA

Release of cinnamate anion from ZLH host against time was measured *in situ* at λ_max_ = 272 nm using a Perkin-Elmer UV–VIS spectrometer Lambda 35 (Perkin-Elmer, Massachusetts, USA) by adding 0.2 mg of sample into 3.5 mL of deionized water, 0.5 mol/L NaCl and pH 5.5 phosphate buffer solution, at room temperature. Data was collected and fitted to zero-, first-, pseudo-second order and parabolic diffusion kinetic models.

### Cell culture

Human dermal fibroblasts were obtained from ATCC (Virginia, USA). Human dermal fibroblast cells were cultured at 37°C and 5% CO_2_ in high glucose Dulbecco’s Modified Eagle Medium (DMEM) (ScienCell Research Laboratories, California, USA) containing 2% fetal bovine serum, 0.5% penicillin–streptomycin, 1% glutamine, and 1% non-essential amino acids. Cultured cells were passaged using 0.25% trypsin. At 85% confluence, cells were harvested using 0.25% trypsin and were subcultured/seeded into 96-well plates.

### Cell viability assay

Healthy human dermal fibroblast cells (at a density of 1 × 10^4^ cells/well) were seeded in a 96-well culture plate. Cultured cells were incubated for 24 h prior to treatment. The cells were growing until 80% confluence, and the media were replaced with different gradient concentrations (from 0.781 μg/mL to 25 μg/mL) of sample in media for 24 h. After the exposure time, sample containing media were aspirated. The cells were then incubated with freshly prepared MTT solution in fresh medium for 4 h at 37°C until a purple colored formazan product developed. After the incubation time, 100 μL DMSO was added to each well to dissolve the formazan crystals. Absorbance of the solution in 96 well plates was measured at 570 and 690 nm using a Bio-Tek ELISA microplate reader EL800 (Vermont, USA). Cell viability was analyzed as a ratio of sample treated cells to untreated cells (control at 0 μg/mL).

## Results and discussion

### X-ray diffraction and spatial orientation of the guest between ZLH interlayers

**Figure 1:**
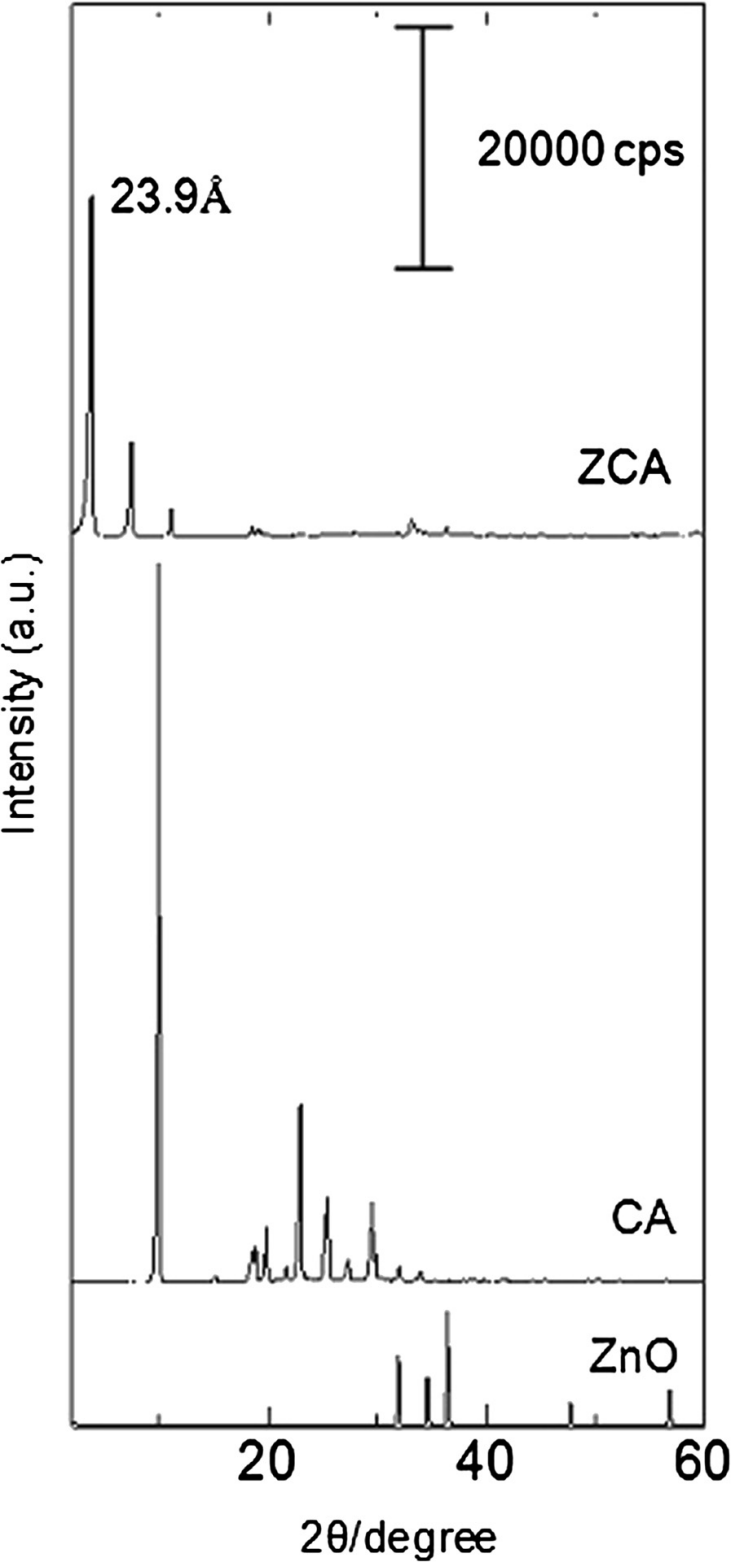
PXRD patterns of ZnO, CA and ZCA intercalation compound.

Figure 1 shows PXRD patterns of ZnO, CA and ZCA intercalation compound synthesized using 0.1 mol/L CA. PXRD pattern of ZnO showed high crystallinity of ZnO characteristic peaks; especially (100), (200) and (101) reflections. We proposed that the formation of cinnamate-ZLH intercalation compound (ZCA) from ZnO occurred in three steps through dissociation-deposition mechanism [6,16-19]. The first step, described in Eq. 1, involves hydrolysis of ZnO in water. When ZnO particles are immersed in water, the surface of ZnO hydrolyzes to form a layer of Zn(OH)_2_. The layer of Zn(OH)_2_ formed then become more soluble than ZnO in the presence of acid to become Zn^2+^ and OH^-^ (Eq. 2). Finally Zn^2+^ species, hydroxyls, H_2_O and cinnamate anions (CA^-^) in the solution react to generate the layered intercalation compound (Eq. 3). The process is repeated until all the ZnO phase and the Zn(OH)_2_ phase has completely converted to the layered compound. The mechanism is described in equations below:

(1) $${\mathrm{ZnO}+\text{H}}_{2}O⇌{\mathrm{Zn}\left(\mathrm{OH}\right)}_{2}$$

(2) $${\mathrm{Zn}\left(\mathrm{OH}\right)}_{2}⇌{\mathrm{Zn}}^{2+}{+2\mathrm{OH}}^{-}$$

(3) $$\begin{array}{c} {\mathrm{Zn}}^{2+}+{2\mathrm{OH}}^{-}+{\mathrm{CA}}^{-}\\ +{\text{H}}_{2}O⇌{\mathrm{Zn}}^{2+}{\left(\mathrm{OH}\right)}_{2-x}{{(\mathrm{CA}}^{-})}_{x}n{\text{H}}_{2}O \end{array}$$

**Figure 2:**
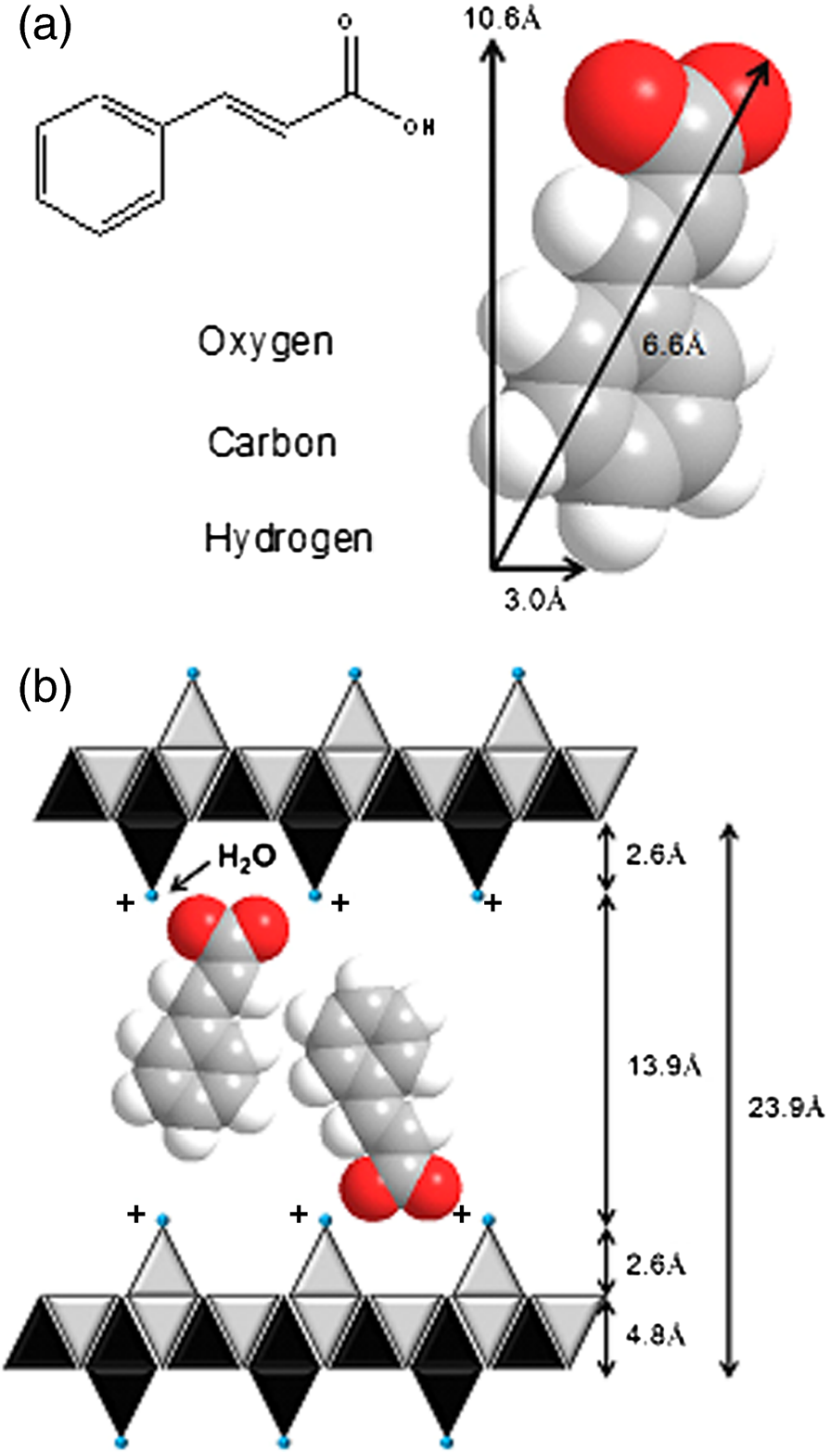
Molecular structure of cinnamate and three-dimensional molecular size of cinnamate (a) and proposed spatial orientation of cinnamate in the interlayer of ZLH (b).

Figure 1(a) shows the PXRD pattern of ZCA synthesized via direct reaction between CA and ZnO. The average basal spacing of the product was measured to be 23.9 Å, based on 3 harmonics. The proposed spatial arrangement of cinnamate within the ZLH interlayer region is based on the basal spacing obtained from PXRD and molecular size of cinnamate anion, as shown in Figure 2(a). Taking into account that the layer thickness is 4.8 Å including 2.6 Å for each zinc tetrahedron [20], the expected gallery height that can be occupied is 13.9 Å. Considering the charge density of the layer, anion dimension and assuming that the layer structure remains intact after the intercalation, then cinnamate anions have to orient themselves in a bilayer arrangement by turning the functional group on the opposite side and opposing the fields of aromatic ring mutually by π-π interactions as shown in Figure 2(b).

Sun *et al.* reported intercalation of cinnamate anion into Zn/Al LDH as host. The reported basal spacing of intercalation compound is 18.0 Å [4]. Subtracting layer thickness, the gallery available to be occupied is 13.2 Å which is relatively close to the value recorded for ZLH host. Hence we can deduce that cinnamate maintains similar orientation in both hosts. However, the observed basal spacing for ZLH host is significantly larger than what was reported for LDH host. We proposed that ZCA intercalation compound follows LHS type IIb structure which is formed by one quarter of the octahedral zinc cations displaced from main layer to tetrahedral sites located above and below each empty tetrahedron. The base of the tetrahedral share hydroxide groups with the octrahedral layer and the apex is occupied by water molecules. Thus, the tetrahedral sheet accounts for larger basal spacing recorded for cinnamate intercalation in ZLH host.

### FTIR spectroscopy

**Figure 3:**
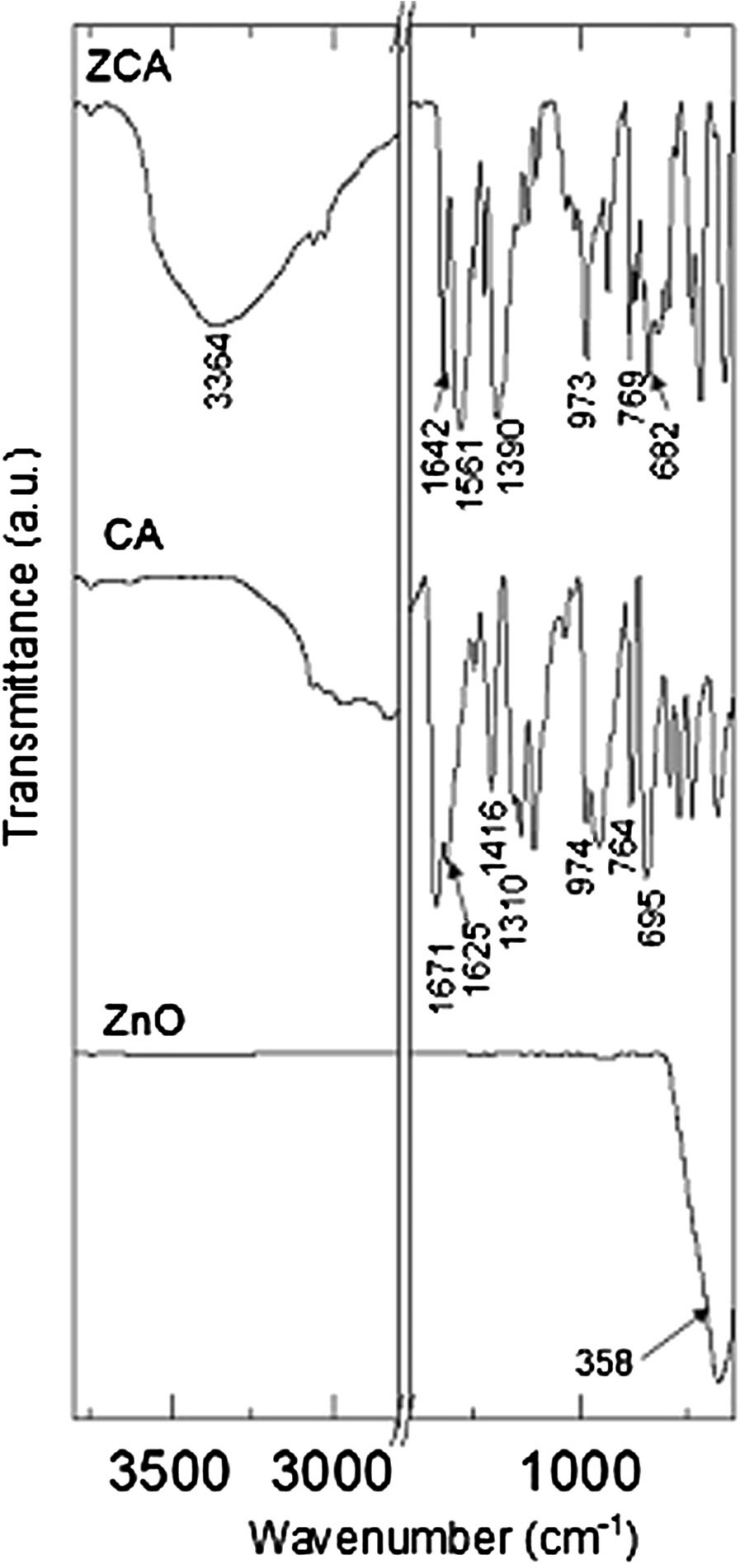
FTIR spectra of ZnO, CA and ZCA intercalation compound.

The FTIR spectra of ZnO, CA and ZCA reacted with 0.1 mol/L CA solution are shown in Figure 3. FTIR spectra of pure ZnO showed a strong peak at 358 cm^-1^ due to vibration of zinc and oxygen sublattices [21]. FTIR spectra of CA showed strong characteristic vibrations at 1671 cm^-1^ attributed to C=O stretching, 1625 cm^-1^ to C=C stretching, 1310 cm^-1^ to C-O stretching, and 1416 cm^-1^ to OH in-plane bending. *Trans*-C-H out-of-plane bend for CA was detected at 974 cm^-1^. C-H monosubstitution band for phenyl group could be detected at 764 cm^-1^ and 695 cm^-1^. On the other hand, the intercalation compound exhibits most of the vibrations assigned to cinnamate, although several vibrations shifted due to interaction between cinnamate anion and the interlayer. In particular, vibrations due to *trans*-C-H out-of-plane bend (973 cm^-1^) and C-H monosubstitution band (769 cm^-1^ and 682 cm^-1^). COO^-^ stretching of intercalated cinnamate anion overlapped and appeared at 1561 and 1390 cm^-1^. Highly overlapped C=C stretch in pure CA is instead more defined as intercalated cinnamate by showing a strong band at 1642 cm^-1^. The broad band at 3364 cm^-1^ is assigned to the O-H stretching of vibration of interlayer water molecules. These results indicate that cinnamate anions are present in the sample and were intercalated between positively charged ZLH layers, as indicated by the characteristics of PXRD pattern.

### Surface properties

**Figure 4:**
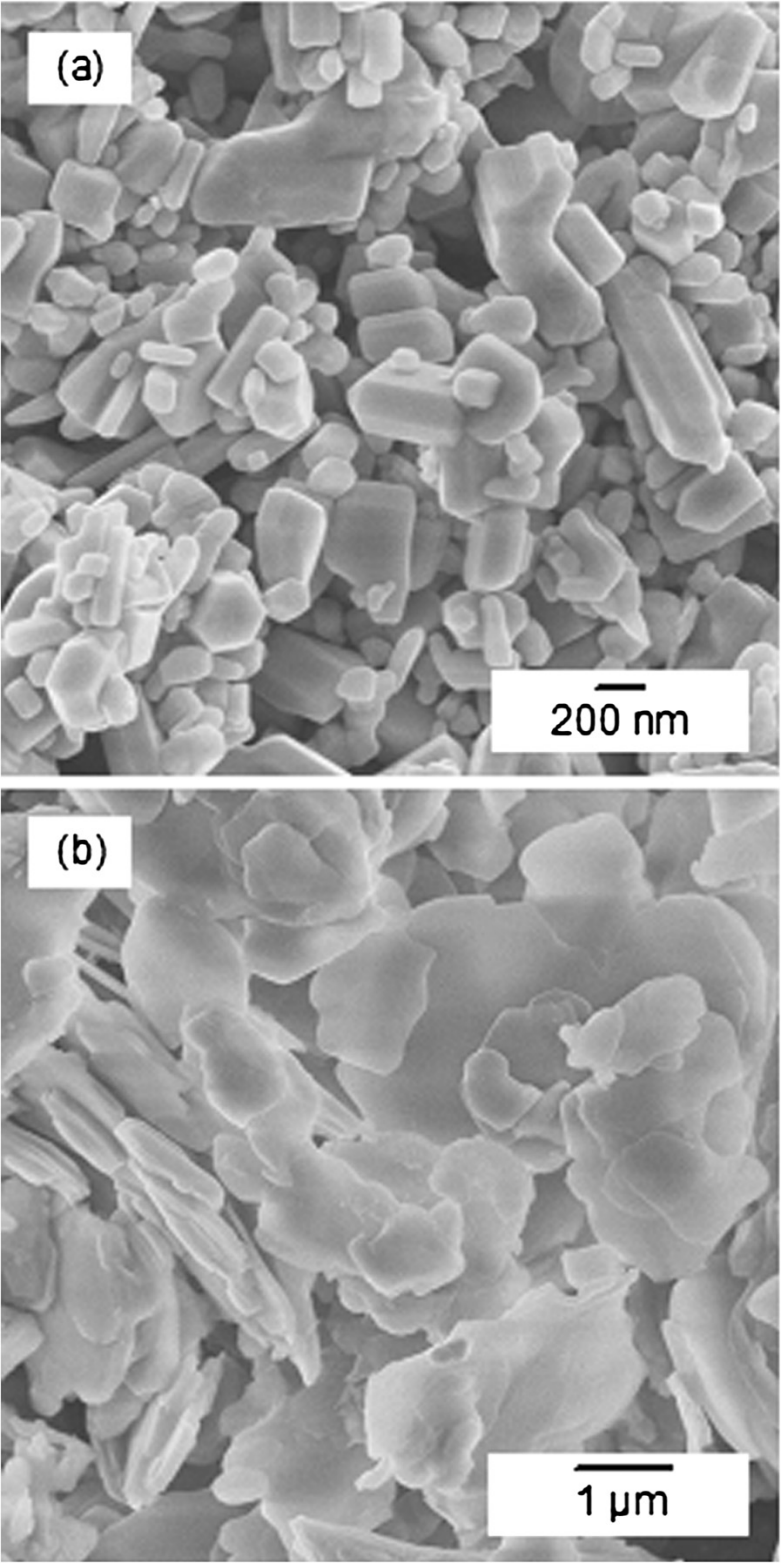
FESEM images of ZnO at 25,000x magnification (a) and ZCA intercalation compound at 10,000x magnification (b).

FESEM images of pure ZnO and ZCA intercalation compound are shown in Figure 4(a) and Figure 4(b), respectively. ZnO is shown to have a granular structure with various shapes and sizes. The size of ZnO nanoparticles ranges from 50–600 nm. The nanoparticles of ZnO are converted to agglomerates of plate-like ZCA particles with non uniform shapes and sizes, in the range of micrometer order. Increase in particle size is evident with the increase of surface area, as recorded in Table 1.

**Table 1:** Physico-chemical properties of ZnO and ZCA intercalation compound

**Sample**	**C (%)**	**H (%)**	^**a**^**Zn (%w/w)**	^**b**^**Anion (%w/w)**	**BET surface area (m**^**2**^**/g)**	**BJH pore diameter (Å)**	**BJH pore volume (m**^**2**^**/g)**
ZnO	-	-	(80.3)^*^	-	5	91	0.01
ZCA	29.5	3.3	38.0^a^v	40.4	13	141	0.05

Notes: ^a^estimated from ICP analysis; ^b^estimated from CHNS analysis; ^*^theoretical value.

ZnO has long been used in sunscreen products as it carries a strong capability to absorb UV rays. However, recent findings shows hair follicle allows penetration of nanoparticles up to 320 nm in size and excitation of ZnO due to sunlight and household items produces oxygen radical species that plays a role in skin aging and photocarcinogenesis [22-24]. In a way, conversion of ZnO precursor to particles of ZCA that are bigger in size will prevent penetration into hair follicles.

**Figure 5:**
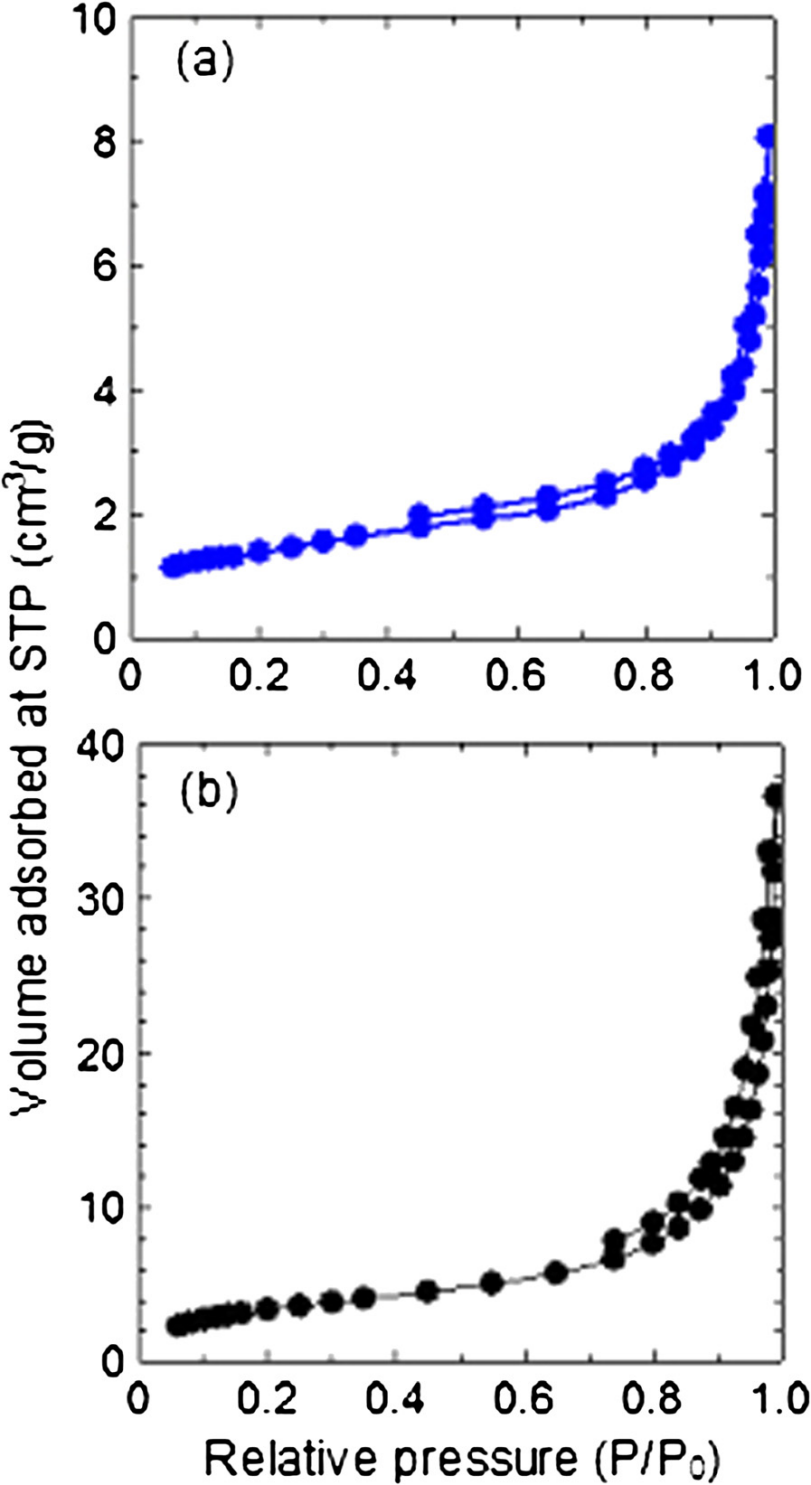
Adsorption-desorption isotherms of ZnO (a) and ZCA intercalation compound (b).

**Figure 6:**
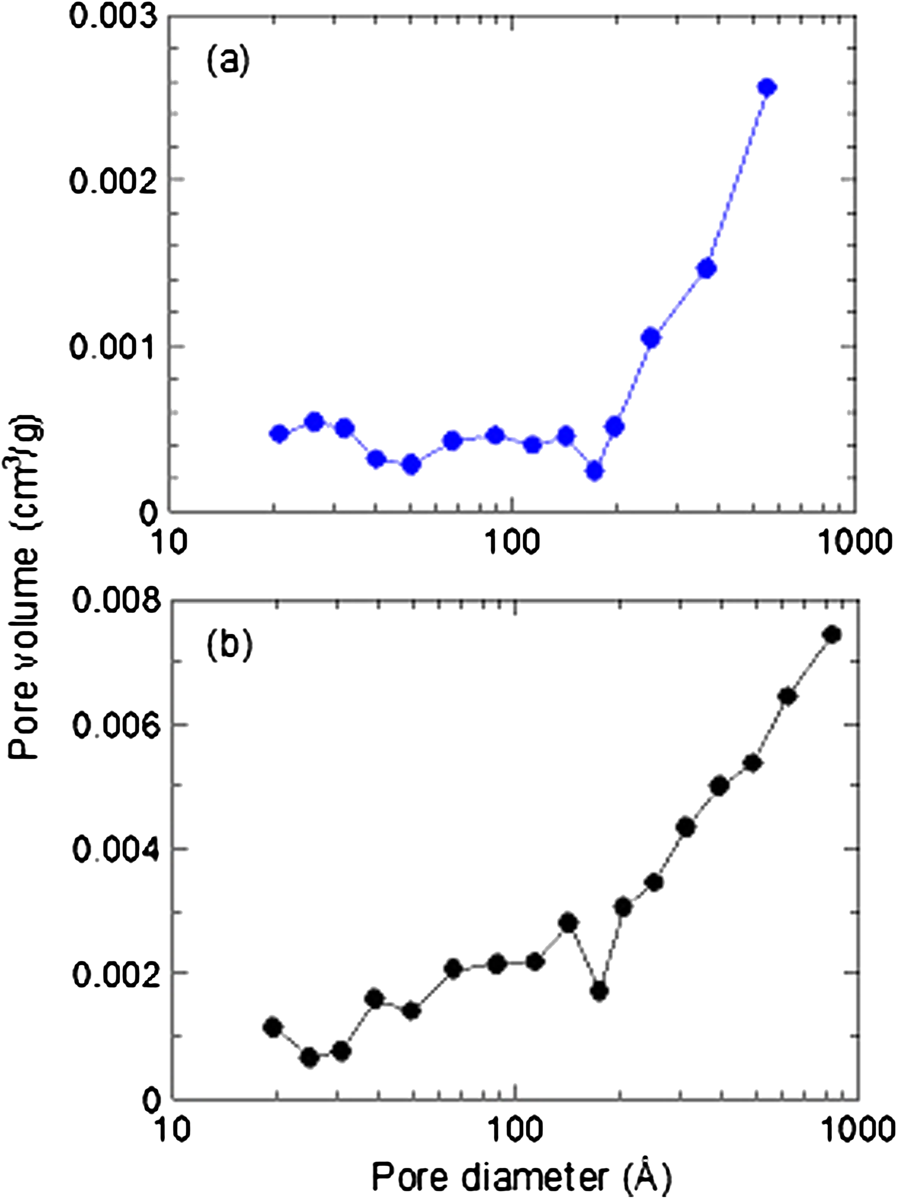
Pore size distribution of ZnO (a) and ZCA intercalation compound (b).

Figure 5(a) and 5(b) show the nitrogen adsorption-desorption isotherms of ZnO and ZCA intercalation compound, respectively. Both precursor and intercalation compound depicts Type IV isotherm with H3-type hysteresis loop by International Union of Pure and Applied Chemistry (IUPAC) classification. This type of loop is typical for mesoporous materials comprised of agglomerates of plate-like particles with slit shaped pores [25]. ZnO showed slow adsorbate uptake at relative pressure range of 0.0 – 0.4 and reaches the optimum uptake at 8 cm^3^/g while ZCA intercalation compound showed rapid adsorption around relative pressure range, 0.0 – 0.7 and reached the maximum adsorption at 37 cm^3^/g. Desorption branch for ZnO is much narrower compared to ZCA. In addition, we recorded an increase in pore size and pore volume from ZnO to ZCA intercalation compound as shown in Table 1. These results indicate a modification of pore texture as a result of transformation from ZnO precursor to layered intercalation compound. FESEM images show a wide distribution of pores for both ZnO (Figure 6(a)) and ZCA intercalation compound (Figure 6(b)).

### Thermal analysis

**Figure 7:**
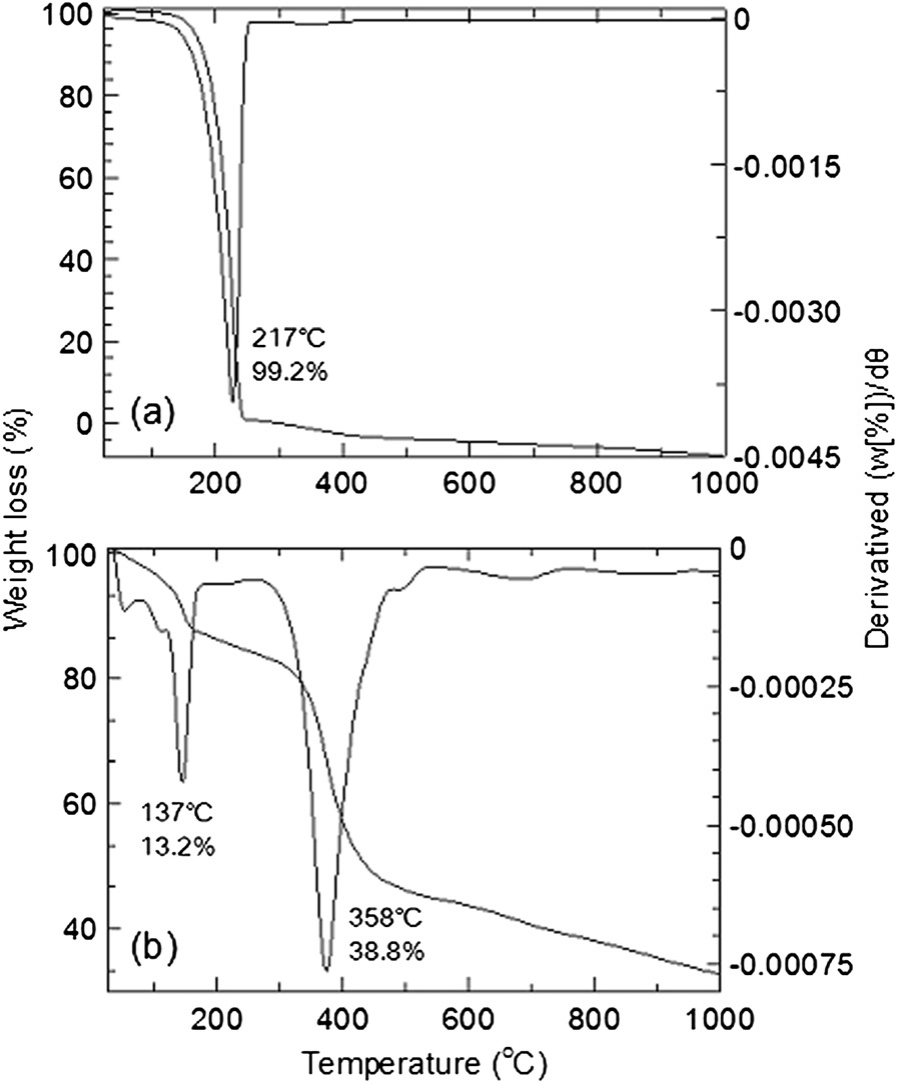
TGA/DTG analyses of CA (a) and ZCA intercalation compound (b).

TGA/DTG measurement of cinnamic acid and ZCA intercalation compound is shown in Figure 7. Thermal analysis curves revealed decomposition profile of pure CA occurred at 217°C (99.2%) in one step while decomposition of ZCA intercalation compound occurred in two steps, the first one is at around 137°C (weight loss of 13.2%) and the second step is at around 358°C (weight loss of 38.8%). The first weight loss attributes to removal of physisorbed and interlayer water. The second weight loss corresponds to the decomposition of intercalated cinnamate. This value is close to the estimated value made from elemental analysis of ZCA, in which it was estimated that the percentage loading of cinnamate in ZLH interlayer to be about 40.4%, as shown in Table 1. The degradation temperature attributing to cinnamate in the intercalation compound is higher than the decomposition of pure CA. This indicates that the thermal stability of intercalated organic sunscreen cinnamate is enhanced due to the interaction with ZLH host.

### Optical properties

**Figure 8:**
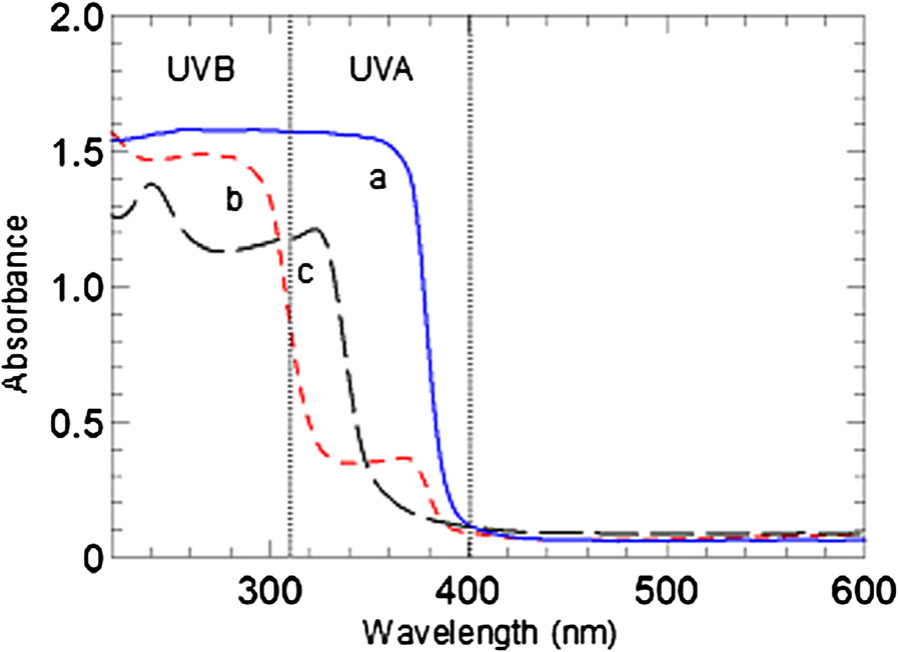
Solid-state absorbance spectra of (a) ZnO, (b) CA and (c) ZCA intercalation compound.

Solid state absorbance spectra of ZnO, CA, and ZLH intercalated with cinnamate anions via direct method are shown in Figure 8. ZnO itself is an excellent UV ray absorbent with high absorption ability that covers both UVA and UVB range. Cinnamic acid is white in colour, with the absorbance peaks at around 240 nm and 325 nm. After intercalation, ZCA intercalation compound showed broadening in absorption range and a shift in absorbance peaks to the higher wavelength region (266–370 nm). Decrease in absorption ability of intercalated sunscreen molecules can be attributed to the dilution effect of confinement in metal hydroxide layers. Broadening of absorption range of intercalation compound is due to spatial confinement and host-guest interactions, namely electrostatic attraction, hydrogen bonding and van der Waals forces. Absorption peak shift to the higher wavelength region (red shift) is due to the edge-to-edge association of intercalated organic sunscreen molecules as proposed in Figure 2[26]. Intermolecular interaction is usually as a result of π-π interactions or hydrogen bonds. As a result of head-to-tail arrangement of cinnamate molecules, electrostatic attraction occurs between dipoles, which resulted in decreased excitation energy and a shift of absorbance band to the higher wavelength region.

**Figure 9:**
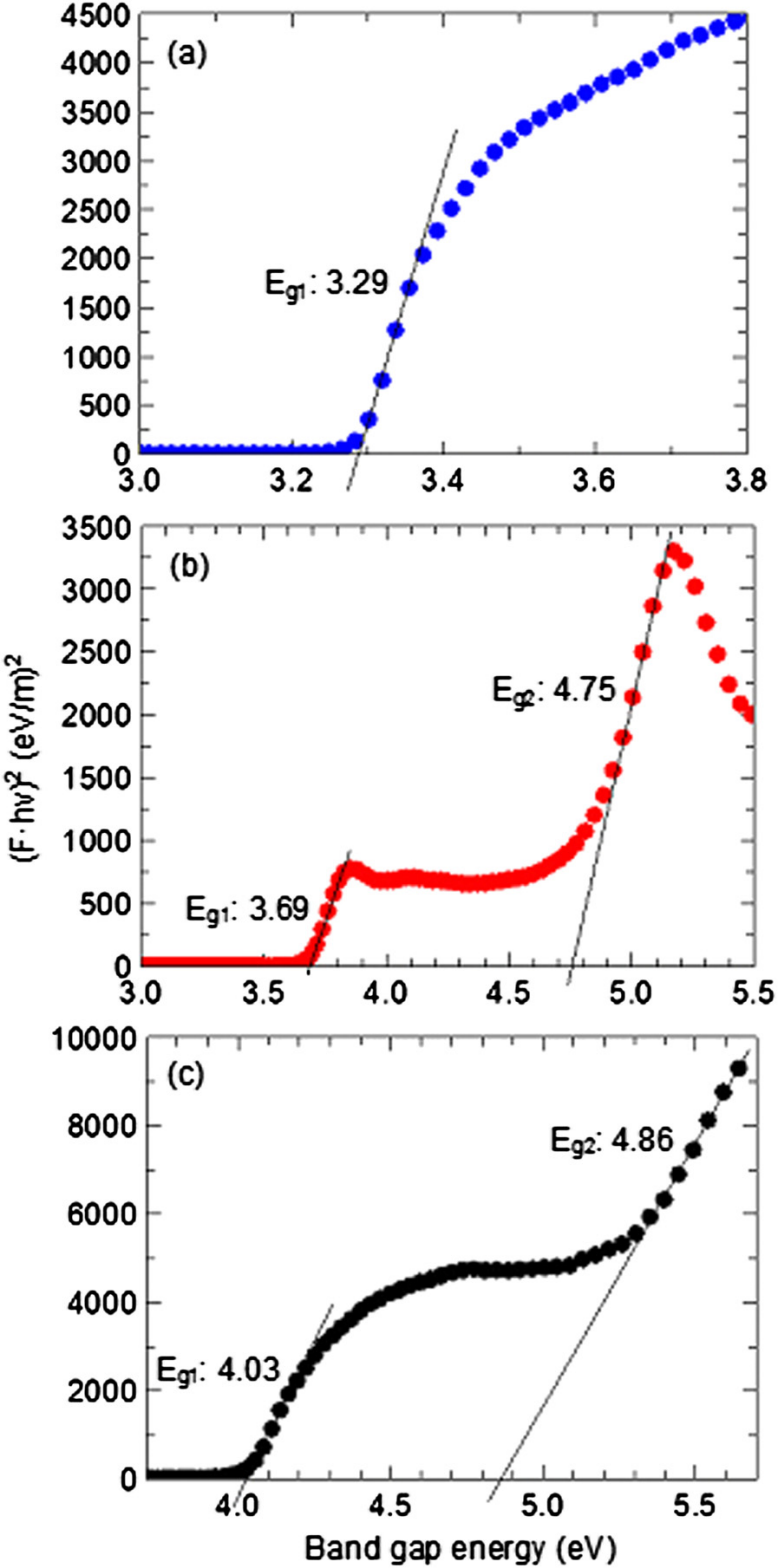
Kubelka-Munk transformed reflectance spectra of (a) ZCA, (b) CA and (c) ZCA intercalation compound.

UV–vis diffuse reflectance spectrum was used to obtain the band gap using the Kubelka-Munk equation [27];

(4) $${\left(\text{F}\cdot \mathrm{hv}\right)}^{2}=A\left(\mathrm{hv}-E_{g}\right)$$

where F is the Kubelka-Munk, h is Planck`s constant, A is a proportionality constant, hv is the photon energy and E_g_ is the band gap energy. Band gap values of samples can be extracted by plotting Eq. 4 as (F·hv)^2^ against hv and extrapolating the linear region straight line to the hv intercept. Band gap of ZnO, CA and ZCA intercalation compound was investigated by these measurements, as shown in Figure 9. Low band gap value for ZnO at 3.29 eV accounts for its high photocatalytic ability. CA showed more than one band gap, at 3.69 eV and 4.75 eV, which is attributed by chromophores in its structure. ZCA intercalation compound maintained the same band gap value as pure CA, as a result of the sunscreen molecule structure being sandwiched between ZLH interlayers. However, the intercalated product showed a shift to a higher band gap at 4.03 eV and 4.86 eV. This is due to the effect of stabilization brought upon immobilization of organic molecules in host. Lower band gaps are a result of higher photocatalytic efficiency due to easier transition from the ground state to the excited state. Reduction in photocatalytic activity in sunscreen is encouraged as to counter production of radical oxygen species, a prominent problem with precursor, ZnO [28,29]. Thus, a shift to a lower photocatalytic efficiency shown by ZCA intercalation compound is highly beneficial.

### Release behavior of cinnamate anions

**Figure 10:**
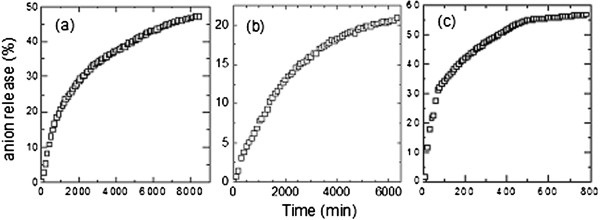
Controlled release of ZCA in deionized water (a), 0.5 mol/L NaCl (b) and pH 5.5 phosphate buffer solution (c).

Release profiles of cinnamate from ZLH matrices in deionized water (a), 0.5 mol/L NaCl (b) and pH 5.5 phosphate buffer (c) are shown in Figure 10. Due to anion exchange capability, intercalated cinnamate could be released and exchanged with anions in release media. We proposed circumstances usually came across with sunscreen application to monitor the stability of our formulation in various conditions. Deionized water was chosen as a control while 0.5 mol/L NaCl and pH 5.5 phosphate buffer was respectively chosen to simulate sea water and pH of skin. The results demonstrated that ZCA reached a saturated released of 47.34% after 6 days in deionized water, 21.07% after 4.4 days in 0.5 mol/L NaCl and 57.16% after 13 h in pH 5.5 phosphate buffer. These results show that our sunscreen molecule is barely released from ZLH interlayer even after it was immersed within extended period of time while most of it remains entrapped inside the inorganic layered host.

ZLH as host for sunscreen molecules will have some level of release as it has anion exchange capability but the release was shown to be very slow and achieved saturation state at low concentration. These results shows novelty of ZLH use in sunscreen formulation as it provides prolonged UV protection as well as prevention of UV ray absorbent molecules photodegradation into toxic degradation products.

### Release kinetics of cinnamate from ZCA intercalation compound

Kinetic release of cinnamate from ZCA intercalation compound was investigated using various kinetic models; zeroth- (Eq. 5) [30], first- (Eq. 6) [31], parabolic diffusion (Eq. 7) [32] and pseudo-second order kinetics (Eq. 8) [33]. The equations are as given below, where *c* is a constant, *C*_*eq*_ and *C*_*t*_ is the concentration of anion at equilibrium and time *t*, respectively.

(5) $$C_{t}=\mathrm{kt}+c$$

(6) $$-\log\left(1-C_{t}\right)=\mathrm{kt}$$

(7) $$C_{t}/C_{\mathrm{eq}}=c+kt^{0.5}$$

(8) $$t/C_{t}=1/k_{2}C_{\mathrm{eq}}{}^{2}+\left(1/q_{e}\right)t$$

**Figure 11:**
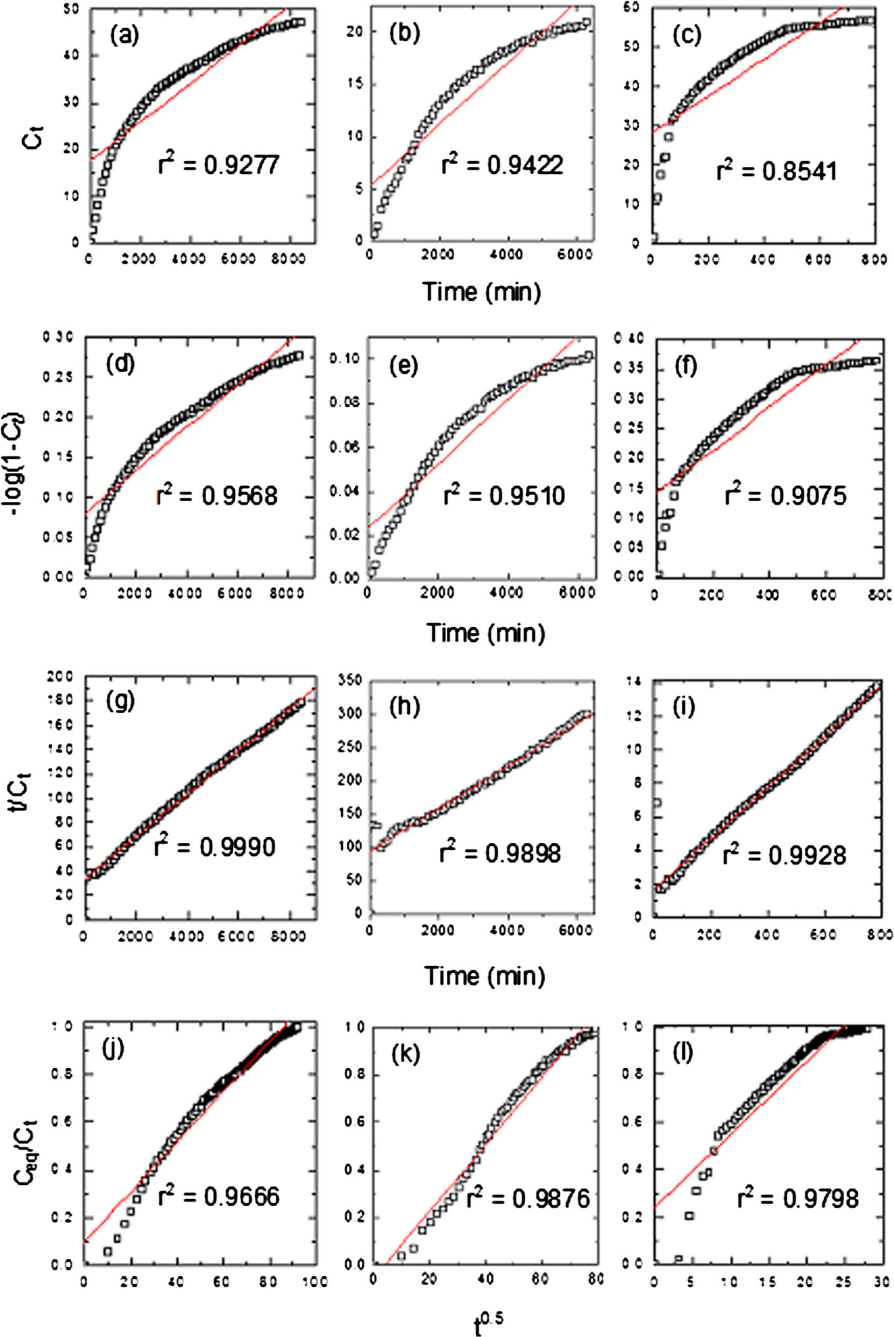
Fitting the data release of CA from ZCA intercalation compound into media for zeroth-, first-, pseudo second order and parabolic diffusion kinetics for deionized water (a, d, g and j respectively), 0.5 mol/L NaCl (b, e, h and k respectively) and pH 5.5 phosphate buffer solution (c, f, i and l respectively).

The plots are given in Figure 11. As evident with the values of correlation coefficient, r^2^ in Table 2, the release of cinnamate anion from ZLH interlayer follows the pseudo-second order kinetic. As a result of the fitting, we calculated that the time taken for cinnamate release to be half of accumulated release, t_1/2_ value, to be 1924 min, 2974 min and 110 min for release in deionized water, 0.5 mol/L NaCl and pH 5.5 phosphate buffer solution, respectively. The t_1/2_ value could be summarized in the order of; phosphate buffer > deionized water > 0.5 mol/L NaCl.

**Table 2:** Correlation coefficient, rate constant and half time obtained by fitting the release data of cinnamate from ZCA intercalation compound into various media using zeroth-, first-, parabolic diffusion and pseudo-second order kinetic models

**Media**	**Saturated release (%)**	**Correlation coefficient, *r***^**2**^				**Rate constant of pseudo second order, *k *(L mg**^**-1 **^**min**^**-1**^**)**	***t***_**1/2 **_**of pseudo second order(min)**
		**Zeroth order**	**First order**	**Parabolic diffusion**	**Pseudo-second order**		
Deionized water	47.34	0.9277	0.9568	0.9666	0.9990	9.09 × 10^-6^	1924
0.5 mol/L NaCl	21.07	0.9422	0.9510	0.9876	0.9898	1.07 × 10^-5^	2974
Phosphate buffer pH 5.5	57.16	0.8541	0.9075	0.9798	0.9928	1.38 × 10^-4^	110

Zeroth order: *C*_*t*_ = *kt* + *c,* first order: −log(1−*C*_*t*_) = *kt* + *c*, parabolic diffusion: *C*_*t*_*/C*_*eq*_ = *c* + *kt*^*0.5*^, pseudo second order: *t/C*_*t*_ = 1/*k*_*2*_*C*_*eq*_^*2*^ + (1/*q*_*e*_)*t.*

Notes: *c* = a constant; *C*_*eq*_ = concentration of anion at equilibrium; *C*_*t*_ = concentration of anion at time *t.*

Release rate of the anion from the ZLH interlayer is influenced by the affinity of available anions in the media. The presence of carbonate, an anion known to have the strongest affinity towards ZLH interlayer, in deionized water was responsible for relatively fast and high accumulated release. Low accumulated release and slow release rate in 0.5 mol/L NaCl is attributed to low ion exchange affinity of chloride towards interlayer of ZLH. Phosphate buffer solution consists of phosphate, carbonate and chloride while 0.5 mol/L NaCl contains only chloride. Release rate and accumulated release will be elevated with the presence of phosphate combined with other anions due to multiple hydrolysis of phosphate [34,35]. ZCA was found to have the highest accumulated release and fastest rate in skin pH simulation. Nonetheless, ZCA reached a saturated release in phosphate buffer after prolonged time (13 h) in comparison to actual use of sunscreen product. Furthermore, pH 5.5 phosphate buffer only reproduces skin pH value and not the real physiological conditions.

### Effect of ZLH-sunscreen intercalation compound on cell viability

The biomedical applications of synthesized nanoparticles are currently attracting much research interest. The progress and application of nanotechnology enhances the quality of our human lives but also results in a health burden. Major issue in determining the efficacy of these materials is assessing their potential cellular toxicity either due to their inherent chemical composition/structure or as a consequence of their nanoscale biophysical properties [36]. In order to evaluate directly if these synthesized nanoparticles were in a range to be physiologically harmful to human skin, we tested their effects on human dermal fibroblasts using a cell viability biological assays.

**Figure 12:**
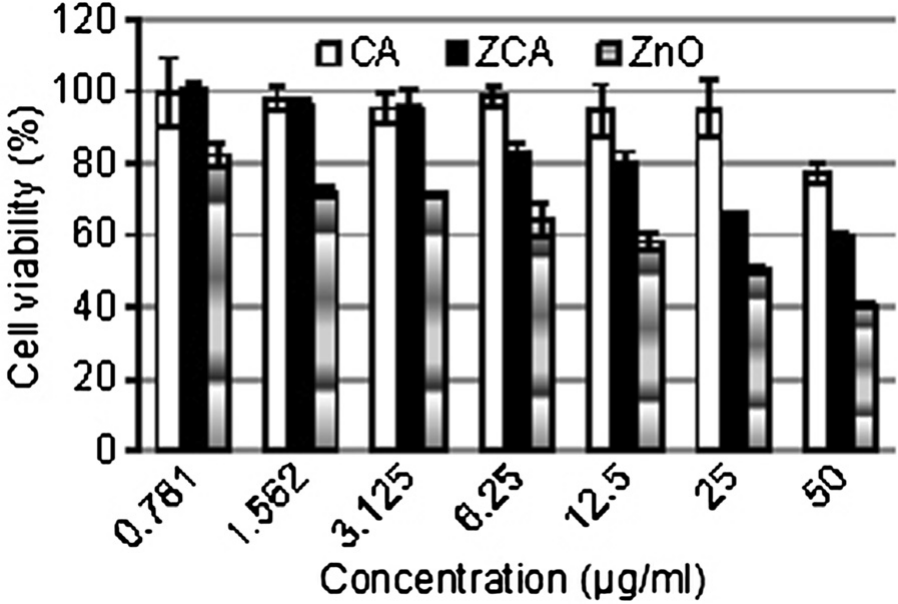
**Cell viability of HDF cells after 24 hours treatment with CA, ZCA and ZnO.** The data were presented as mean ± S.D.

Human dermal fibroblast cells were exposed to samples at various gradient concentrations of 0.781, 1.562, 3.125, 6.25, 12.5, 25 and 50 μg/mL for 24 h, and cell cytotoxicity was evaluated by MTT assay (Figure 12). ZCA intercalation compound at the concentration from 0.781 to 12.5 μg/mL did not show any noticeable reduction in cell viability. Cells exposed to 25 and 50 μg/mL of intercalation compound showed around 30% and greater than 50% reduction, respectively in cell viability (Figure 12).

We found that synthesized intercalation compound exposure effectively reduced cell viability of human dermal fibroblasts at concentration 25 μg/mL and above. Based on this finding we suggest that intercalation compound dosage up to 12.5 μg/mL did not produce any cytotoxicity. Hence, further studies should focus at the range of the concentration not more than that 25 μg/mL, to develop the cosmetic product using the particular nanocarrier. At a concentration higher than 25 μg/mL, substantial study to focus on dermal toxicity with experimental animals for translational studies to provide systematic molecular mechanisms for biomedical application.

## Conclusions

In the present work, organic UV-ray absorbing active agent, cinnamate anion has been successfully intercalated into ZLH interlayers spaces from zinc oxide precursor to generate ZCA intercalation compound with a basal spacing of 23.9 Å to accommodate cinnamates in a bilayer arrangement. ZCA intercalation compound retained excellent absorption capacity in the UV region of pure CA but with slight shift in absorption peaks and broadened absorption range due to arrangement in host and host-guest interactions. Retention of cinnamate in ZLH interlayers was tested with various media to show slow release and saturated release at a very low concentration. Hence it was demonstrated that the resulting material is suitable to be used as sunscreen with long term UV protection effect.

## Abbreviations

ZLH: Zinc layered hydroxide;CA: Cinnamic acid;ZnO: Zinc oxide;HDF: Human dermal fibroblast;UV: Ultraviolet;LHS: Layered hydroxide salt;ZCA: Cinnamate- zinc layered hydroxide intercalation compound;BET: Brunauer-Emmett-Teller;BJH: Barrett-Joyner-Helenda

## Competing interests

The authors declare that they have no competing interests.

## Authors’ contributions

SMNM produced samples, performed data analysis and interpretation and drafted the manuscript; MZH conceived the study, participated in the design and coordination of scientific teams and assisted to draft the manuscript; SHS assisted in the design of cinnamate retention study; SF participated in the design of cytotoxicity study; PA carried out the MTT assay and interpretation of cytotoxicity results; TYH assisted in X-ray diffraction and interpretation. All authors read and approved the final manuscript.

## Authors’ information

Prof. Dr. Mohd Zobir Hussein is a Professor of Chemistry in Institute of Advanced Technology (ITMA), Universiti Putra Malaysia. His major research areas include layered organic–inorganic nanohybrid for gene and drug delivery, nanoparticles and nanostructured materials, their design, synthesis and applications. He is a prolific author and has contributed to more than 200 technical papers. He is the assignor of 1 granted patent on the preparation method of nanomaterial for controlled release formulation and co-assignor of another 2 granted patents.
